# Effective Vaccine Management: The Case of a Rural District in Ghana

**DOI:** 10.1155/2019/5287287

**Published:** 2019-10-13

**Authors:** Eric Osei, Mohammed Ibrahim, Gregory Kofi Amenuvegbe

**Affiliations:** ^1^Department of Population and Behavioral Sciences, School of Public Health, University of Health and Allied Sciences, Ho, Ghana; ^2^Bolgatanga Municipal Health Directorate, Ghana Health Services, Upper East Region, Bolgatanga, Ghana; ^3^Department of Health Policy, Planning, and Management, School of Public Health, University of Health and Allied Sciences, Ho, Ghana

## Abstract

**Background:**

The Effective Vaccine Management (EVM) initiative provides the platform needed to monitor and assess the vaccine supply chain system to identify strengths and weaknesses of the system at all levels to enhance the development of improvement plan to strengthen the system. This valuation was carried out in the Tolon District of the Northern Region, Ghana.

**Methods:**

A descriptive valuation of vaccine management was carried out in six vaccine stores in the Tolon District of Northern Ghana. We employed World Health Organization (WHO) assessment tools and procedures which consisted of desk reviews and interviews of cold chain managers to assess vaccine management practices in the district. Five out of the nine global assessment criteria were assessed and a minimum target level required for all criteria to meet the WHO standard was 80%.

**Results:**

None of the facilities assessed met the WHO benchmark of 80% for all but one criteria assessed. With regards to temperature control, the scores ranged from 42% at Kasuliyili CHPS Centre to 77% at the district store with an average district score of 60%. Stock management ranged between 11% at Wantugu Health Centre and 75% at Nyankpala Health Centre with district average score of 32%. Effective vaccine distribution scores ranged between 13% at Kasuliyili CHPS and 46% at Nyankpala Health Centre with an average district score of 27%. Only Nyankpala Health Centre had an acceptable score of 84% for vaccine management, whereas the lowest score for this indicator was 5% at Tolon Health Centre store with district average score of 53%. Information management and supportive functions scores ranged from 0% at Tolon Health Centre to 26% at the district store with the district average score of 16%. Nineteen (90.5%) of vaccine users had poor knowledge regarding temperature control and vaccine distribution.

**Conclusion:**

Effective vaccine management knowledge and practices are poor at Tonlon district and calls for urgent and pragmatic approaches such as training and re-training of vaccine users at all levels.

## 1. Background

The Expanded Programme on Immunization (EPI) has proved its place as a corner stone in the Primary Health Care (PHC) strategy. Not only is it a cost-efficient intervention that prevents common childhood diseases, it also provides an entry-point into communities for other Reproductive and Chid Heath (RCH) interventions, such as vitamin A supplementation and growth promotion [1].

Immunization programmes depend greatly on efficient and effective supply chain systems to store, transport and distribute these vaccines and health commodities, which ensures that the right products are available at the right place, at the right time and in the right condition in order to provide efficient health services to the communities [2]. Evidence have shown that effective vaccine supply chain system is one of the most vital elements of any immunization programme, which ensures that vaccines reach recipients in their potent form [2].

The Effective Vaccine Management (EVM) initiative, launched in the year 2010 by World Health Organization (WHO) and United Nations Children Fund (UNICEF) is a comprehensive assessment of the vaccine supply chain system of immunization programmes in low and lower-middle income countries [3]. The focus of the initiative is to uncover the shortcomings in the performance of immunization supply chain so as to provide basis for improvement [3]. EVM initiative assesses nine criteria of vaccine management performance from the national through to the service delivery points and benchmark this performance against global set standards. The nine criteria for satisfactory vaccine supply chain are; (i) vaccine arrival procedures—ensure that every shipment from the manufacturer reaches the national store in right condition; (ii) temperature monitoring—vaccines and diluents are stored and distributed within recommended temperature ranges; (iii) cold storage, dry storage, and transport capacity—warrant the sufficiency of storage and transportation of all vaccines and supplies needed for the immunization programme; (iv) buildings, cold chain equipment, and transport systems are appropriate; (v) maintenance of buildings, cold chain equipment, and vehicles; (vi) stock management systems; (vii) vaccine distribution between each level in the supply chain; (viii) implementation of appropriate vaccine management policies are adopted; and (ix) satisfactory information systems and Supportive management functions [3, 4]. Each of the nine criteria is assessed at each level of the supply chain—from the national through to the service delivery level by observation, inspection of infrastructure and records, and by interview of health staff involve in vaccine handling and management [3].

In Ghana, two nationwide EVM assessments were conducted in selected districts in September 2010 and October 2014. The results showed that temperature monitoring, which is a critical indicator for vaccine potency was poor at the national level and even poorer at the regional and health facility levels [4]. These results spawned concern and interest from stakeholders and called for a critical appraisal of the results and also expand the assessment to identify the contributing factors considering Ghana's advancement in immunizations [4]. This study was therefore conducted in Tolon District of the Northern Region of Ghana, to evaluate the effectiveness of the vaccine supply chain system to address these concerns.

## 2. Materials and Methods

### 2.1. Study Setting

**Figure 1:**
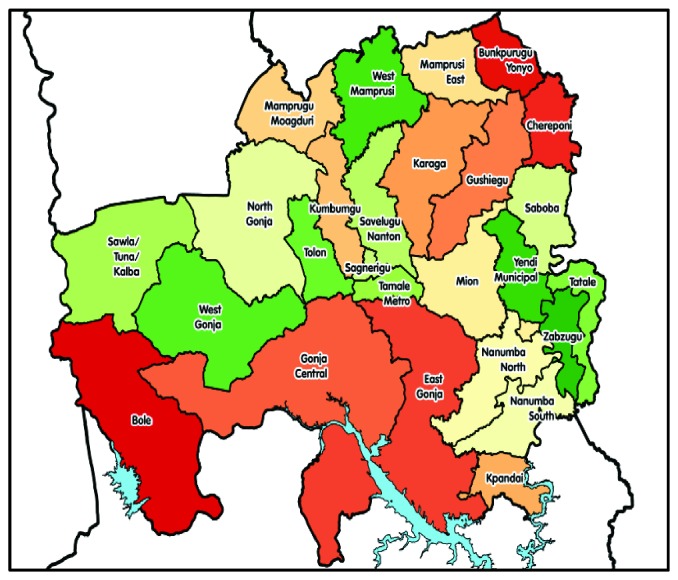
Map of Northern Region.

The assessment was conducted in Tolon District of the Northern region of Ghana in April 2017. Tolon district is one of the rural districts in Northern Ghana created in the year 2011. The population of the District according to the 2010 Population and Housing Census, is 72,990 representing about 2.9% of the region's total population. Almost ninety percent (88.4%) of the district population is rural and more than 73% of the population are not literate [5]. There are three Health Centres, ten Community-based Health Planning and Services (CHPS) Centres and a private Clinic. The only hospital in the district is privately owned. The district vaccine store, three health centres stores (Nyankpala H/C, Wantugu H/C, and Tolon H/C), and two Community-based Health Planning and Services (CHPS) stores [Kpendua CHPS and Kasuliyili] were purposefully sampled and assessed. These facilities were those with vaccine storage facilities and stored vaccines [6].  Figure 1 depicts the map of Northern Region of Ghana showing the study district.

### 2.2. Study Design and Population

A facility based descriptive cross-sectional study that employed WHO's EVM assessment tools and procedures [7] was conducted to assess vaccine management knowledge and practices. In this study, five of the nine criteria develop by WHO were valuated. These criteria were; (1) temperature control: to assess that vaccines and diluents are stored within the WHO recommended temperature ranges in the cold chain system, (2) stock management: to assess that effective stock management systems and procedures are in place, (3) distribution: to assess that vaccines are distributed between each level in the supply chain in an effective manner, (4) vaccine management: to assess that appropriate vaccine management policies are adopted and implemented at all levels of the immunization supply chain, and (5) information systems: to assess that relevant information systems and supportive management functions are satisfactory. All health personnel who handle and use vaccines were identified and interviewed. Among this population were Disease Control Officers, Enrolled Nurses, and Community Health Nurses.

### 2.3. Instrument and Data Collection

The study was conducted in April, 2017 using an EVM assessment tool developed by WHO and UNICEF in 2010 [7]. The tool is a Microsoft Windows based application used to describe the supply chain system and contain standard questions in Excel workbooks for each level of the supply chain—from national to service delivery level. It sets standards in nine criteria of vaccine management based on well-established principle and standards for quality management. The tool was administered to health staffs responsible for vaccine management at all levels. Observations of the cold chain and desk review of related documents were done to complement data collected through interviews. Among the variable assessed were, monitoring of cold chain quality, accuracy of temperature recording devices, record keeping of temperature records, recording and reporting of stock transactions, maintenance of vaccine stock levels, periodic physical inventory, warehouse practices, management of short shipments, monitoring of quality of the distribution system, knowledge and practice of vaccine handling, availability of SOPs and annual work plans, training and supervision of key management staff and collection and use of data for decision-making. In addition to the EVM tool, a pre-tested questionnaire was designed and administered to all public health staff who handle and use vaccines in their service delivery to assess their general knowledge regarding vaccine management. Trained health staff with experience in vaccine management collected the data and an average of two hours was spent to collect data from each participating facility.

## 3. Data Analysis

Data were entered into the EVM assessment tool for analysis. The tool is designed in such a way that once the necessary data are entered; it generates performance indicators and criteria scores for individual facilities assessed—for each level of the supply chain and for the entire supply chain. Every criterion is given a score out of 100% and the minimum acceptable score for each criterion at each level of the supply chain is set at 80% by WHO in order to be regarded as effective and reliable [8]. Any score less than 80% is marked italics and scores above 80% is marked bold in this study indicating acceptable performance.

Data captured using the questionnaire to assess knowledge of other staff regarding vaccine handling were entered into Epi Data Version 3 and exported to STATA Version 12 for analysis. The total questions were 38, covering Vaccine Vial Monitor (VVM), shake test, Multi-dose vaccine vial policy (MDVVP), ice packs conditioning, supply period, temperature monitoring, and immunization waste management. Thus, the level of knowledge was graded in accordance with the number of correct answers (“YES”) the individual scored out of the 38 items. Each correct answer to an item was scored as one and wrong answer was scored as zero. The scale was categorized into poor knowledge, moderate knowledge, and good knowledge. Any respondent who scored between 0 and 15 questions was considered as having poor knowledge, scoring between 16 and 30 was considered as having moderate knowledge and scoring 31 and above was considered as having good knowledge in vaccine handling and management.

### 3.1. Ethical Issues

The study was approved by the Ghana Health Service Ethical Review Committee with reference No. GHS-ERC/35/10/16. Permission to undertake and publish the study was obtained from the District Director of Health Services and the heads of the facilities of the district. Written informed consent was sought from all participants. All the study participants were clearly informed about the objectives or purposes, procedures, risk and benefits, privacy, and confidentiality of the study before data collection.

## 4. Results

### 4.1. Characteristics of Study Participants

Of the six facilities assessed, a total of 21 health staff were interviewed. Of these, 16 (76%) were females and the rest were males. The majority of them (13; 62%) were between the ages of 24 and 29 years, 7 (33.3%) between 30 and 35 years, and 1 (5%) above 36 years old. Fifteen (71%) of them were married and 6 (29%) never married. With regards to category of staff, 12 (57%) were Community Health Nurses, 4 (19%) Disease Control Officers, and 5 (24%) Enrol Nurses. Nine (43%) of the respondents had between 1 and 3 years working experience, 10 (47%) had worked for 4–6 years, and 2 (10%) had served for more than 7 years (Table 1).

### 4.2. Summary of Consolidated EVM Indicator Scores

**Figure 2:**
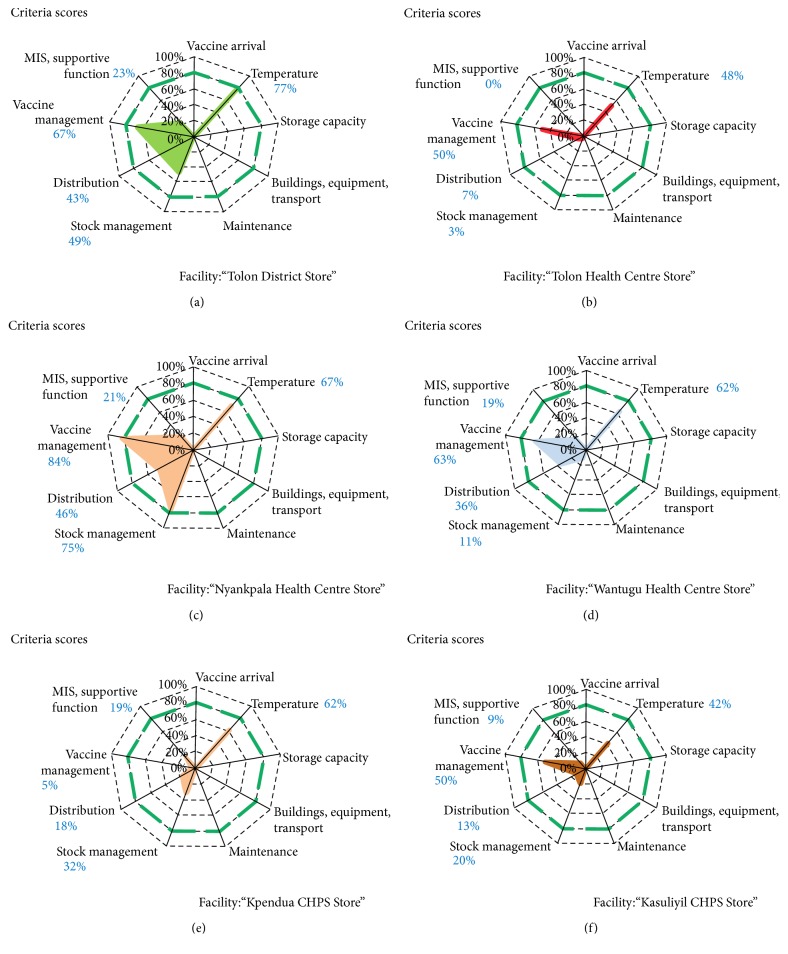
Resulting spider web showing supply chain performance by facility.

**Figure 3:**
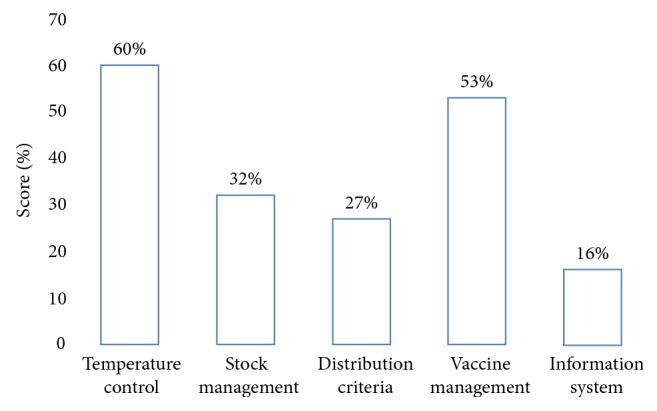
District average score of effective vaccine management.

Table 2 and Figure 2 show summaries of consolidated results. WHO recommends a minimum of 80% of performance for each criterion. Hence, scores less than 80% are marked in italics to accentuate the need for attention whereas scores of 80%–89% are left in the normal font to depict that they are in the acceptable range and 90% and above are marked in bold to indicate best performance. The areas covered by the polygons in Figure 2 indicate the achievements in the areas of the respective indicators. All facilities assessed could not meet the 80% benchmark for all indicators and hence in italics marks except for Nyankpala Health Centre that scored an acceptable 84% for vaccine management. Temperature control scores ranged from 42% in Kasuliyili CHPS to 77% in Tolon District store with district average of 60%. Vaccine stock management scores ranged from 3% in Tolon Health Centre store to 75% in Nyankpala Health Centre store, while vaccine distribution scores ranged between 7% and 46%. The range of scores for vaccine management and Information system and supportive functions were 5%–84% and 0–26% respectively. The district average scores were 60%, 32%, 27%, 53%, and 16% for Temperature control, stock management, distribution, vaccine management, and Information system respectively (Figure 3).

### 4.3. General Knowledge of Vaccine Handlers on Temperature Control and Vaccine Distribution

#### 4.3.1. Temperature Control

All the 21 health personnel interviewed have heard of Vaccine Vial Monitor (VVM), however, 13 (61.9%) out of these could tell its purpose, 8 (38.1%) could explain how VVM works, and 5 (23.8%) knew its important. Twelve (57%) of respondents have heard of the “shake test.” Of these, 3 (25%) knew the prerequisite for shake test and could demonstrate how the test is done correctly. Of the 21 respondents interviewed, 4 (19%) could mention all vaccines that are sensitive to freezing and knew the recommended storage temperature for these categories of vaccines. Five (23%) of them have heard of “conditioning” ice packs. Of these, 1 (20%) knew the purpose for conditioning ice packs for vaccine transportation and could demonstrate correctly how ice packs are conditioned. Ten (47.6%) of respondents have heard of the multi dose vaccine vial policy. Of these, 3 (30%) knew the period within which open vials can be reused as stipulated in the policy. Seven (33.3%) out of the 21 respondents could explain how to defrost vaccine fridge correctly (Table 3).

#### 4.3.2. Vaccine Distribution

Of the 21 respondents, 6 (29%) have heard of “vaccine supply period.” Of these, 2 (33.3%) knew the purpose of the supply period and could tell correctly the vaccine supply period for their facility store. Three (14.3%) of the respondents have heard of FIFO/FEFO/EEFO rule and could tell when the rule should be applied in vaccine distribution (Table 3). Overall, none of the respondent had the requisite knowledge in temperature control and vaccine distribution, 2 (9.5%) had moderate knowledge and 14 (90.5%) had poor knowledge.

## 5. Discussion

Adequate vaccine management and safety practices is required at all levels of the supply chain and at all times in order to achieve to goals of immunization programmes [3]. This criterion seeks to ensure that all recommended policies for vaccine management are adopted and implemented effectively at all levels, including the use of vaccine vial monitors (VVMs), the “shake-test,” the multi-dose vial policy (MDVP), and the monitoring of vaccine wastages [3]. The substandard vaccine management practices at all levels observed in this current study corroborates the result of previous assessment in Ghana, where even national and regional levels could not meet the acceptable scores of 80% [4]. This has negative impact on storage conditions of vaccines, which can compromise the safety and potency of the vaccines [9] and therefore calls immediate and pragmatic measures to deal with it. Vaccine management is both managerial and operational functions that seek to ensure that adequate and high-quality vaccines are readily available for immunization service delivery [10]. Hence, any inadequacies of this critical function will lead to interruptions of vaccine supply. In 2014 for instance, vaccine stock outs were reported in about one-third 31% of low and lower-middle-income countries at the national level and in 26% at the district level [11] leading to “missed” opportunities and inequitable access to life saving vaccines. The absence of written policies and procedures pose vaccines to inappropriate handling and storage conditions even in the developed world such as the United Kingdom [2]. Therefore, protocols for ordering, storing and handling of vaccines should be available at the workplace and to ensure that health workers use them in order to improve on vaccine management practices.

Vaccines are biological products that can lose their potency when exposed to both heat and freezing, and hence are require to be stored within strict temperature ranges in a cold chain system [10, 12]. However, none of the facilities assessed in this study met the benchmark of 80% for temperature control. Temperature control is the responsibility of all vaccine handlers and hence ought to have requisite knowledge to perform this duty [13, 14]. Knowledge regarding temperature control such as the application of Vaccine Vial Monitor (VVM), the shake test, and the multi-dose vial policy among the health staff studied was also generally inadequate and could contribute to the poor temperature control practices observed. This finding is similar to assessments in Tanzania [15] and Cameroon [16]. The need for urgent redress of the knowledge gaps in temperature control if the vaccine supply chain must function at the required standard cannot be over emphasized in order that vaccine safety and potency are not compromised. Previous EVM evaluation in Ghana identified poor temperature control practices even at the national level and identified lack of training and continues temperature monitoring devices, inadequate knowledge in vaccine management among others as contributory factors [17]. WHO identify in 2014 that only 14% in low and lower-middle-income countries met the standard criteria for temperature control in the cold chain [10]. Global action is therefore required to address the challenge of ensuring that vaccine recipients receive potent and save vaccines in order to achieve immunization goals.

Vaccine stock management and procedure is another key component of the vaccine supply chain system, which seeks to ensure that vaccine handling, physical inventory, stock-control systems, stock-level management, good warehousing practice, and disposal procedures for damaged and expired vaccines are done in accordance to global standards [18]. Effective stock management in this study was poor at all levels and even abysmal the service delivery levels. The average district score for stock management was only 32%. If proper vaccine forecasting, and other stock management procedures are not properly maintained, it could interrupt the supply of vaccines and injections equipment, which will lead to many children left unvaccinated. This current finding is comparable to what the International Federation of Pharmaceuticals Manufacturers and Associations (IFPMA) reported in their 2014 study where less than one-fourth of countries are operating at a minimum Effective Vaccine Management (EVM) levels for maintenance and stock management [19]. The bottlenecks of effective vaccine stock management need to be identified and addressed at all levels of the supply chain otherwise, the efforts of immunization programmes will be jeopardized, limiting access to, and use of vaccines [20].

Distribution of vaccines between and within levels of the supply chain system seeks to ensure that the transportation of vaccines and other commodities are done in an effective manner, including the correct use of passive containers (cold boxes), appropriate packing practices, and maintaining transport contingency plans [21]. In the current study, vaccine distribution was found to be unsatisfactory at all facilities assessed with a district average of just 27%. This could be as a result of logistical, financial, and human resources constrains [21]. The knowledge gap identified among health staff regarding vaccine distribution in this study and the bad nature of most roads in the country, particularly the Tolon District, resulting in long hours of transporting vaccines from one place to the another may have implication on distribution of vaccines.

Effective information systems and supportive management functions, including the use of standard operating procedures and supportive management functions are critical for effective functional of the immunization programmes [22]. Performance of this criterion was woefully inadequate in all the facilities assessed. This affects the vaccine needs as well as other necessary resources, and may have negative impact on the immunization programme, since it can lead to poor vaccine forecasting, avoidable stock outs, and poor management of vaccine wastage. The result of the recent study is consistent with a similar assessment done by UNICEF in Gujarat–India in 2011, which reported an average facility score of 11% in information systems and supportive management functions [18]. The result is also comparable to what was reported in a study in 2013 in developing countries, which suggest managerial oversight in effective vaccine management has been largely neglected and underestimated [13]. Managers need accurate and timely information that allows them to verify vaccines and equipment needs, validate vaccine coverage, and identify weak links in the supply chain in order to develop measures to address these. Managers must learn how to make information available and use the information to forecast vaccine needs, allocate stock, manage staff and resources, modify delivery routes and frequencies, act rapidly where equipment becomes dysfunctional, and recommend policy changes. Just like other emergency services in public health delivery, vaccine handling must be classified as such and be given similar attention by all stakeholders.

The most obvious limitation of this study is its small sample size. Therefore, firm conclusions about the relationships among variables cannot be drawn. However, the study involved all vaccine handlers in each of the facilities assessed, which makes generalization possible.

## 6. Conclusion

Organization and management of vaccines and supplies for immunization in Tolon district is woefully inadequate and calls for urgent and pragmatic measures to address this. Our findings suggest for the need to train and retrain health staff involved in the vaccine supply chain in order to bridge the knowledge gap. This will ensure that all vaccines that reach the recipients are potent in order to achieve immunization programme goals. We recommend further, a region-wide assessment for all nine EVM criteria in order to understand the broader picture of the supply chain in the region.

## Figures and Tables

**Table 1:** Demographic characteristics of respondents.

Background	Frequency (*N* = 21)	Percent (%)
*Age (years)*		
24–29	13	61.9
30–35	7	33.3
36+	1	4.8
*Sex*		
Males	5	23.8
Females	16	76.2
*Marital status*		
Never married	6	28.6
Married	15	71.4
*Staff category*		
Disease Control Officers	4	19.0
Community Health Nurses	12	57.1
Enrolled Nurses	5	23.8
*Number of years in service*		
1–3	9	42.9
4–6	10	47.6
7+	2	9.5

**Table 2:** Summary of consolidated EVM criteria scores by facility, Tolon District.

#	Criteria	Facility scores					
		TD	TCH	NHC	WHC	KPCHPS	KCHPS
1	Temperature control	*77%*	*48%*	*67%*	*62%*	*62%*	*42%*
2	Stock management	*49%*	*3%*	*75%*	*11%*	*32%*	*20%*
3	Distribution	*43%*	*7%*	*46%*	*36%*	*18%*	*13%*
4	Vaccine management	*67%*	*50%*	**84%**	*63%*	*5%*	*50%*
5	Information system	*26%*	*0%*	*21%*	*19%*	*19%*	*9%*

TD: Tolon Distrct; THC: Tolon Health Centre; NHC: Nyankpala Health Centre; WHC: Wantugu Health Centre; KPCHPS: Kpendua Chps; KCHPS; Kasuliyili Chps.

**Table 3:** Knowledge of vaccine users regarding temperature control and vaccine distribution.

Variable	Frequency *n*(%)
*Knowledge on temperature control*	
Have heard of vaccine vial monitor (VVM)	21 (100)
Know the purpose of VVM	13 (61.9)
Could explain how VVM works	8 (38.1)
Know the importance of VVM position on vaccine vial	5 (23.8)
Have heard of the vaccine “shake test”	12 (57.1)
Know the condition requiring “shake test”	3 (25.0)
Could demonstrate the shake test correctly	3 (25.)
Could name vaccines that are sensitive to freezing	4 (19.0)
Know the recommended storage temperature for freeze-sensitive vaccines	4 (19.0)
Have heard of conditioning ice packs	5 (23.8)
Know the purpose for conditioning ice packs	1 (20.0)
Could demonstrate how ice packs are conditioned	1 (20.0)
Have heard of multi-dosed vaccine vial policy	10 (47.6)
Know the period within which open vials can be stored and reuse	3 (30.0)
Could demonstrate how to defrost vaccine fridge	7 (33.3)
*Knowledge on sufficient vaccine distribution*	
Have heard of “vaccine supply period”	6 (29.0)
Know the supply period in the facility	2 (33)
Know the purpose of the supply period	2 (33)
Have heard of FIFO/FEFO/EEFO rule in vaccine distribution	3 (14.3)
Know when to apply FIFO/FEFO/EEFO rule	3 (14.3)

FIFO: first in first out; FEFO: first expiry first out; EEFO: early expiry first out.

## Data Availability

The data used to support the findings of this study are available from the corresponding author upon reasonable request.
